# Multicenter Clinical Validation of an Artificial Intelligence Diagnostic Classification Model for Laryngoscopy Images

**DOI:** 10.1002/ohn.70153

**Published:** 2026-02-16

**Authors:** Claudio Sampieri, Francesco Mora, Giorgio Peretti, Marc Larrosa, Isabel Vilaseca, Francesc X. Avilés‐Jurado, Alessandro Ioppi, Elisa Bellini, Berta Alegre, Laura Ruiz‐Sevilla, Rakesh Srivastava, Athanasios C. Sakellaridis, Andriana Razou, Georgios P. Kotsis, Sara Moccia, Leonardo S. Mattos, Chiara Baldini

**Affiliations:** ^1^ Department of Experimental Medicine (DIMES) University of Genoa Genova Italy; ^2^ Department of Otolaryngology Hospital Clínic Barcelona Spain; ^3^ Unit of Head and Neck Tumors Hospital Clínic Barcelona Spain; ^4^ Unit of Otorhinolaryngology–Head and Neck Surgery IRCCS Ospedale Policlinico San Martino Genova Italy; ^5^ Department of Surgical Sciences and Integrated Diagnostics (DISC) University of Genoa Genova Italy; ^6^ Surgery and Medical‐Surgical Specialties Department, Faculty of Medicine and Health Sciences Universitat de Barcelona Barcelona Spain; ^7^ Department of Otorhinolaryngology‐Head and Neck Surgery S. Chiara Hospital, Azienda Provinciale per i Servizi Sanitari (APSS) Trento Italy; ^8^ Otorhinolaryngology Head‐Neck Surgery Department Hospital Universitari Joan XXIII Tarragona Spain; ^9^ Department of ENT & Head Neck Surgery Sushrut Institute of Plastic Surgery & Super specialty Hospital Lucknow India; ^10^ Department of Otorhinolaryngology/Head & Neck Surgery Saint Savvas Cancer Hospital Athens 11522 Greece; ^11^ Department of Innovative Technologies in Medicine And Dentistry Università degli Studi “G. d'Annunzio’ Chieti Pescara Italy; ^12^ Department of Advanced Robotics Istituto Italiano di Tecnologia Genoa Italy; ^13^ Dipartimento di Informatica, Bioingegneria, Robotica e Ingegneria dei Sistemi (DIBRIS) University of Genoa Genoa Italy

**Keywords:** Artificial Intelligence, head and neck, laryngeal cancer, laryngology, otolaryngology

## Abstract

**Objective:**

To develop and externally validate a computer‐aided diagnosis (CADx) model using artificial intelligence (AI) for classifying laryngeal lesions from laryngoscopy images into high‐risk (HR), low‐risk (LR).

**Study design:**

Retrospective multicenter development of a CADx model and external validation on independent cohorts.

**Setting:**

Multicenter tertiary referral hospitals (Italy, India, China, Greece, and Spain).

**Methods:**

Over 20,000 images derived from laryngoscopic examinations were retrieved. Images were annotated based on histopathology or expert consensus. A deep learning model was trained using an internal dataset and evaluated on 2 external datasets to assess generalizability. The CADx model classifies only images containing visible lesions, discriminating between LR and HR categories. Diagnostic performance was measured using standard metrics, including accuracy, precision, recall, F1‐score, and area under the receiver operating characteristic curve (AUC). Model performance was compared with physicians of varying expertise and ChatGPT‐4o.

**Results:**

The computer‐aided diagnosis model achieved a similar performance across internal and external datasets in distinguishing HR from LR lesions, with accuracy/AUC of 0.90/0.89 internally, 0.85/0.85 on the Greek dataset, and 0.88/0.88 on the Spanish dataset. The model's accuracy was statistically noninferior to that of otolaryngologists and expert laryngologists, and superior to general practitioners and ChatGPT‐4o.

**Conclusion:**

This is a large multicenter clinical validation of a CADx model for laryngeal endoscopy, demonstrating generalizability and performance comparable to clinicians in discriminating between LR and HR lesions. The model's success supports its potential role in augmenting diagnostic capabilities, especially in resource‐limited settings. A prospective multicenter clinical trial is underway to assess real‐world clinical implementation.

Laryngeal lesions are the most common type of disease encountered in the upper aerodigestive tract. The standard examination for diagnosing laryngeal conditions is laryngoscopy using rigid or flexible endoscopes. Notably, recent technological advancements have led to tremendous improvements in endoscopic image quality through high‐definition digital imaging.

Bioendoscopy filters such as Narrow Band Imaging (NBI) have enhanced the diagnostic capability of White Light (WL) endoscopy. NBI is an optical technique that uses spectral light to highlight submucosal vascularization. By enhancing the visualization of neoangiogenic changes, this technique helps otolaryngologists distinguish normal from cancerous or dysplastic tissues.^1^ However, this technology is highly subjective and requires an extensive learning curve to be mastered effectively.^2^ Considering that laryngeal lesions are extremely heterogeneous, the gold standard for diagnosis continues to be biopsy, which is often performed invasively under general anesthesia, with consequent high costs and delays in the diagnosis.

Recent progress in Artificial Intelligence (AI) has the potential to revolutionize the diagnosis of upper aerodigestive tract lesions. Through computer vision techniques, AI can enable computers to derive meaningful information from digital images and videos. This technology is already widely applied in the medical field, achieving remarkable results.^3^ Particularly, AI‐based computer vision methods are already integrated into commercial endoscopic systems for gastroenterology, supporting clinicians in recognizing suspicious lesions during the examination.^4^, ^5^, ^6^ This possibility of performing an optical biopsy with the support of AI is called Computer‐Aided Diagnosis (CADx).^7^

Regarding otorhinolaryngology, computer vision has only recently begun to be explored. Although the literature on CADx is growing, few studies have tested on external populations or validated their results by comparing them to those of human physicians for whom this technology is intended to support. This hampers a clear assessment of the real potential implications of this technology in clinical practice.

With the present study, we aimed to provide a more comprehensive evaluation of CADx systems in laryngology, using a dataset of WL and NBI laryngoscopy images from different centers around the world. With this dataset, we developed an AI‐based CADx model capable of distinguishing High‐Risk (HR) lesions from Low‐Risk (LR) lesions. This model was then tested on external cohorts to assess its generalizability. Finally, we compared the performance of the new CADx model to that of medical professionals. To our knowledge, this is the first worldwide multicenter study with a systematic external validation and clinical assessment.

## Methods

### Data Acquisition

The TRIPOD + AI checklist is available in Supplemental Figure S1, available online. A retrospective study was conducted to retrieve laryngeal images and related histopathological data from the electronic databases of patients from several centers around the world between 2014 and 2023. No study protocol was previously published, and no previous registration was performed due to the retrospective nature of the study. Images were selected according to the availability of pre‐treatment recorded videolaryngoscopies in local databases.

The following datasets were collected, all at tertiary referral centers for laryngeal cancer:

‐ ***Italian*** dataset: collected at the Otolaryngology of Hospital San Martino (Genova, Italy) with local Ethics Committee approval (CER Liguria 230/2019), it comprises 8777 images collected from 889 different patients.
‐ ***Indian*** dataset: collected at the Laryngology unit of the Sushrut Institute of Plastic Surgery & Super Specialty Hospital (Lucknow, India) with local Ethics Committee approval, it comprises 1728 images collected from 960 patients.
‐ ***Spanish*** dataset: collected at the Otolaryngology Unit of Hospital Clínic (Barcelona, Spain) with local Ethics Committee approval (Reg. HCB/2023/0897), it comprises 150 images collected from 150 patients.
‐ ***Greek*** dataset: collected at the Otolaryngology Unit of the Oncologic Hospital Saint Savvas (Athens, Greece) with local Ethics Committee approval, it comprises 909 images collected from 133 different patients.
‐ ***Chinese*** dataset: collected at the Department of Otorhinolaryngology of the Sixth Medical Center of PLA General Hospital (Beijing, China). It comprises 3057 images from 1950 patients, of which only 1489 non‐healthy case images were selected. This is a publicly available dataset by Yin et al called “Laryngoscope8.”^8^

The videos collected at all participating centers were recorded during normal clinical practice using a flexible or rigid high‐definition video‐endoscope (Olympus) capable of both WL and NBI. From these videos, images were manually extracted and annotated to build the datasets mentioned above. Expert physicians with a minimum of 5 years' experience in laryngology and NBI image interpretation performed this task at each clinical center.

Patients without previous treatment on the larynx were included. The image selection process included both WL and NBI imaging modalities and various viewing angles. Images were carefully chosen to ensure the selection of good quality frames providing a clear visualization of the larynx without artifacts such as blur or saliva. The images were annotated using an internal custom‐made computer vision annotation software designed to allow the physicians to easily annotate the images with just a bounding box and the possibility to choose the histologic label without uploading data to an external/online third‐party platform. Annotations were exported in COCO (Common Objects in Context) JSON format. Each laryngeal image was marked with a Bounding Box (BB) encompassing the entire lesion's visible surface and labeled according to its histological type. The lesion label was based on final postoperative histopathological confirmation. The software required a confirmation of the BB by a second physician to validate the annotation. Images that were not validated due to inconsistent labeling by the 2 otolaryngologists were reviewed by author CS, who confirmed one of the 2 annotations or discarded the image if questionable.

The possible lesion types included 8 categories: cyst, granuloma, leukoplakia, polyp, papilloma, Reinke's edema, healthy, and invasive carcinoma. Leukoplakia (consisting of keratosis with several degrees of dysplasia up to in situ carcinoma) and invasive carcinoma lesions were subsequently grouped as HR lesions, while all the others were grouped as LR lesions. Laryngeal frames characterized by the absence of such lesions were organized into a single category named “Healthy.” These annotations are referred to as *ground truth*.

An additional dataset collected for this work was the *Pre‐Training* dataset, comprising 8651 frames automatically extracted from the videos of the same patients included in the *Italian* dataset. This process considered only good‐quality frames identified using the AI model described in the paper by Baldini et al.^9^ In addition, only one frame out of 10 consecutive frames was extracted. These images were automatically labeled considering the overall diagnosis linked to the specific video, and used as an in‐domain pre‐training strategy to enhance the model's performance without requiring manual labeling from physicians.

Finally, the *Italian*, *Indian*, and *Chinese* datasets were combined to create the *Internal* dataset, which was utilized for training and internal testing of the CADx model. The *Greek* and *Spanish* datasets served as 2 *External* datasets for assessing the CADx model's generalization capabilities.

### Deep Learning Model Development

To develop the AI model, and following standard AI methodology, the *Internal* dataset was first reorganized into 3 different sets for training (70%), validation (20%), and testing (10%). This division followed a patient‐level strategy not to have images from the same patients in both the training and testing sets, following standard practice to avoid overfitting.

Then, instead of following a general‐domain pre‐training method (ie, using the ImageNet dataset), this process was based on our custom *Pre‐Training* dataset. After this initial step, the *Internal* dataset was used to fine‐tune the CADx model, enabling the ability to distinguish between LR and HR lesions.

In line with previous studies,^10^, ^11^, ^12^ both model training and inference were performed using frames cropped around the lesion based on the coordinates of annotated bounding boxes. This approach has previously been shown to enhance the performance of the classification algorithm by giving it a region of interest (ROI) to analyze instead of the entire image. In fact, the CADx model was designed to work in a pipeline with our previously published detection model.^13^ The latter flags the lesion on the image and feeds the image within the BB to the CADx model, which ultimately analyzes the cropped image. Consequently, both the training and test sets were annotated with BBs. The cropped images were also resized using a zero‐padding operation to 224 pixels × 224 pixels to ensure consistency as the model's architecture uses predefined input resolutions to balance computational efficiency and accuracy. In addition, data augmentation was used to increase the training dataset size by 30%. This was achieved through image transformations: rotations up to 10°, shift range up to 0.1 in both directions, shear up to 10%, zoom in the range [0, 0.1], horizontal flipping, and random brightness changes.

The deep learning model selected for this work was EfficientNetV2S, one of the most used image classification algorithms, which was chosen considering the trade‐off between efficiency and efficacy. Model training used the following parameters: 100 epochs, batch size of 8, Adam optimizer with an initial learning rate of 1e−4, and exponential decay at a rate of 0.96. Model implementation was conducted using Python and TensorFlow libraries on an NVIDIA Tesla V100 GPU with 16 GB of memory.

### Outcome Analysis

**Figure 1:**
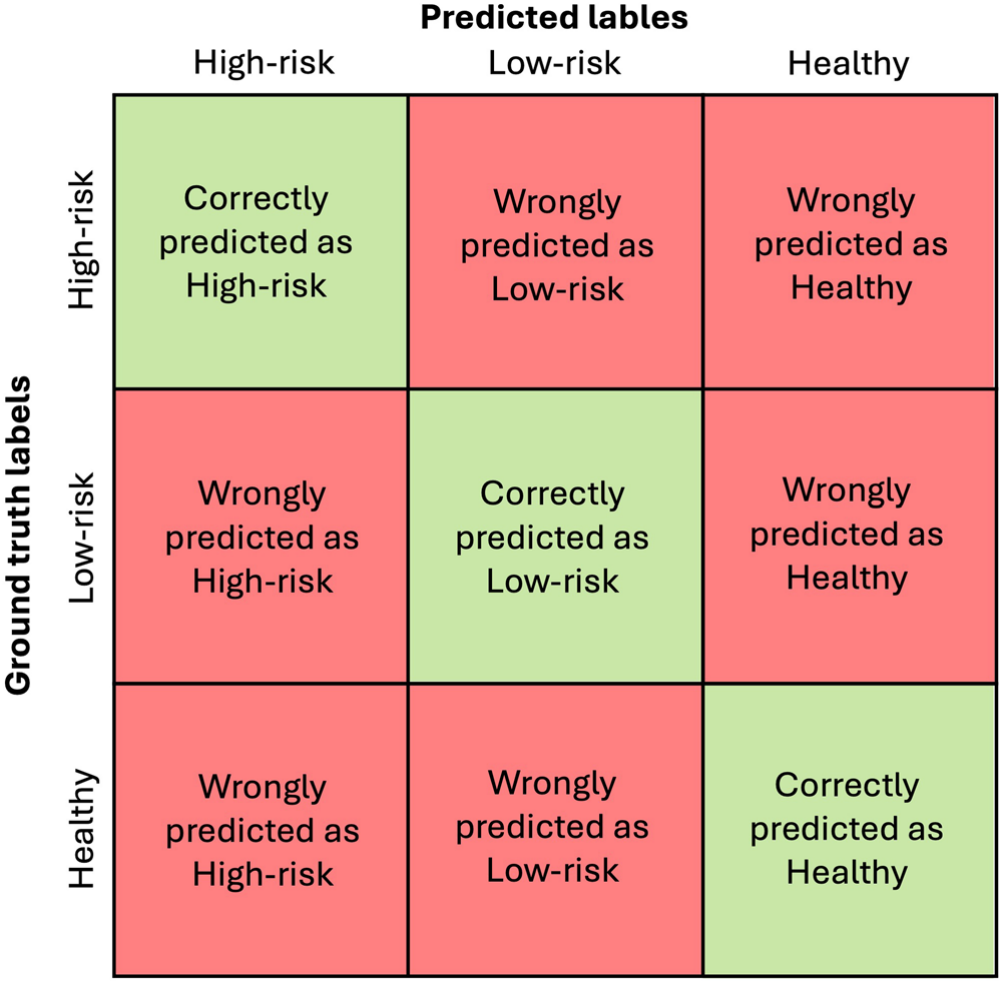
Confusion matrix of model's/rater's predicted instances across 3 categories. The actual labels are on the vertical axis, and the predicted labels are on the horizontal axis. The diagonal cells contain correctly classified instances.

The performance was evaluated by comparing the predicted classes with the ground‐truth, resulting in confusion matrices shown in Figure 1. For every class, a confusion matrix with task‐specific definitions of true positive (TP), true negative (TN), false positive (FP), and false negative (FN) was used.

Since the CADx model was specifically trained to distinguish between LR and HR classes, and the Barcelona dataset included healthy cases without annotated bounding boxes (BB), these images were processed using a previously developed lesion detection system^13^ to extract potential classifier inputs. The image's final prediction was considered healthy if no detection occurred. As the CADx model was trained only to distinguish between LR and HR, and the healthy cases represented only a small subset (n = 12; 8%), whose classification depended on the detection algorithm (whether a lesion was detected or not), they were excluded from the classification metrics computation.

The outcomes of this study were also analyzed using standard diagnostic metrics commonly used in the medical image analysis field.^14^ The explanation of every metric is reported in the supplemental material.

### Comparison of the AI Model With Physicians

To interpret the performance of the CADx model, a comparison with healthcare professionals was performed. For this evaluation, several physicians were selected from the Hospital Clínic of Barcelona. Among the physicians, 3 were senior laryngologists with more than 10 years of experience in laryngoscopy, 3 were general otorhinolaryngologists, 3 were otorhinolaryngology senior residents with 3 to 4 years of training, and 3 were general practitioners with limited contact with laryngoscopy. None of the physicians had been previously exposed to any case in the test dataset. The 150‐image *Spanish* dataset was used for this purpose. Plain images without BB were shown to the raters, and they were asked to classify each image as HR (lesions suggestive of leukoplakia or carcinoma), LR (all other benign lesions), or healthy (no lesion detected). For each group of physicians, the most common class selected by its members for every case was recorded as the final answer for that group.

### Comparison With ChatGPT

**Figure 2:**
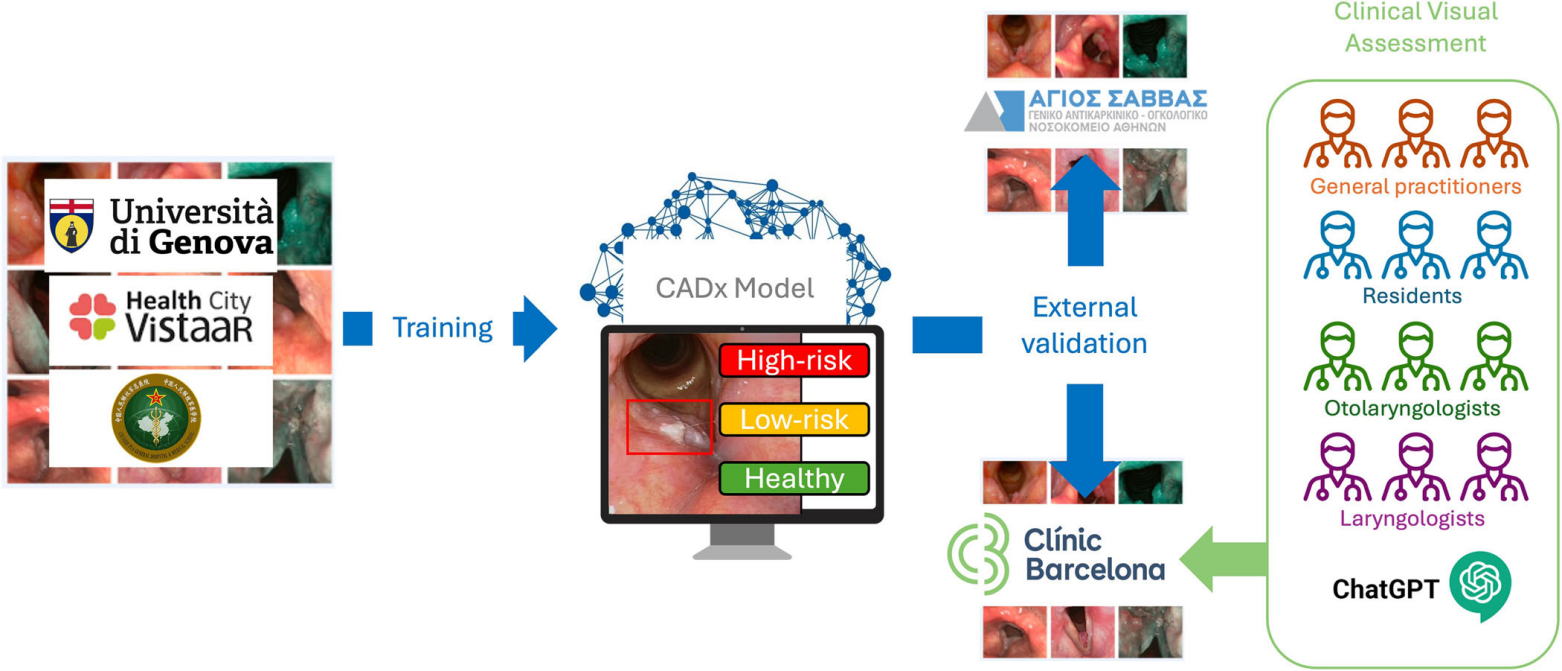
The Genova, Indian, and Chinese datasets were used for training. The CADx model was tested on 2 external datasets: *Greek* and *Spanish*. On the latter, physicians and ChatGPT‐4o rated images for clinical comparisons.

As there have already been some attempts in the literature to use large language models for this same task,^15^ and to evaluate whether our model is better than the state‐of‐the‐art publicly available approaches, we also tested the *Spanish* dataset on ChatGPT‐4o (September 2024). The latter was tested without previous training with the following English prompt: “If I show you some laryngeal endoscopy images, could you tell me which is the diagnosis among these conditions: laryngeal papillomatosis, Reinke's edema, granuloma, polyp, cyst, leukoplakia, carcinoma or healthy larynx.” The images were submitted one by one, and each answer was collected. A scheme summarizing the whole experiment is reported in Figure 2.

### Statistical Analysis

Performance comparisons based on Accuracy metrics between the CADx model and physicians were conducted using McNemar's Test. To statistically compare the Receiver Operating Characteristic (ROC) curves between the internal and the 2 external datasets, pairwise comparisons of the areas under the curve (AUCs) were conducted using a *Z*‐test.^16^, ^17^ 95% Confidence Intervals (CI) were obtained through a bootstrap strategy. Statistical significance was set at *P* < .05. Statistical analyses were performed using Python version 3.9 and R Studio version 2024.09.1+394.

## Results

### Dataset Overview

The total number of frames used for the CADx model development was 21,834, consisting of 13,184 frames cropped based on the annotations and 8651 unlabeled full‐size frames. The *Internal* dataset included 12,118 cropped frames, which were split into 8477 for training, 2415 for validation, and 1226 for testing. The *External* dataset included a total of 1079 images, 909 from the *Greek* dataset, cropped into 929 frames, and 150 from the *Spanish* dataset, of which 13 healthy cases were considered only for the comparisons with physicians and ChatGPT. Demographics were not prospectively recorded during dataset collection, and due to the anonymization of the images, these data were not retrospectively available.

**Figure 3:**
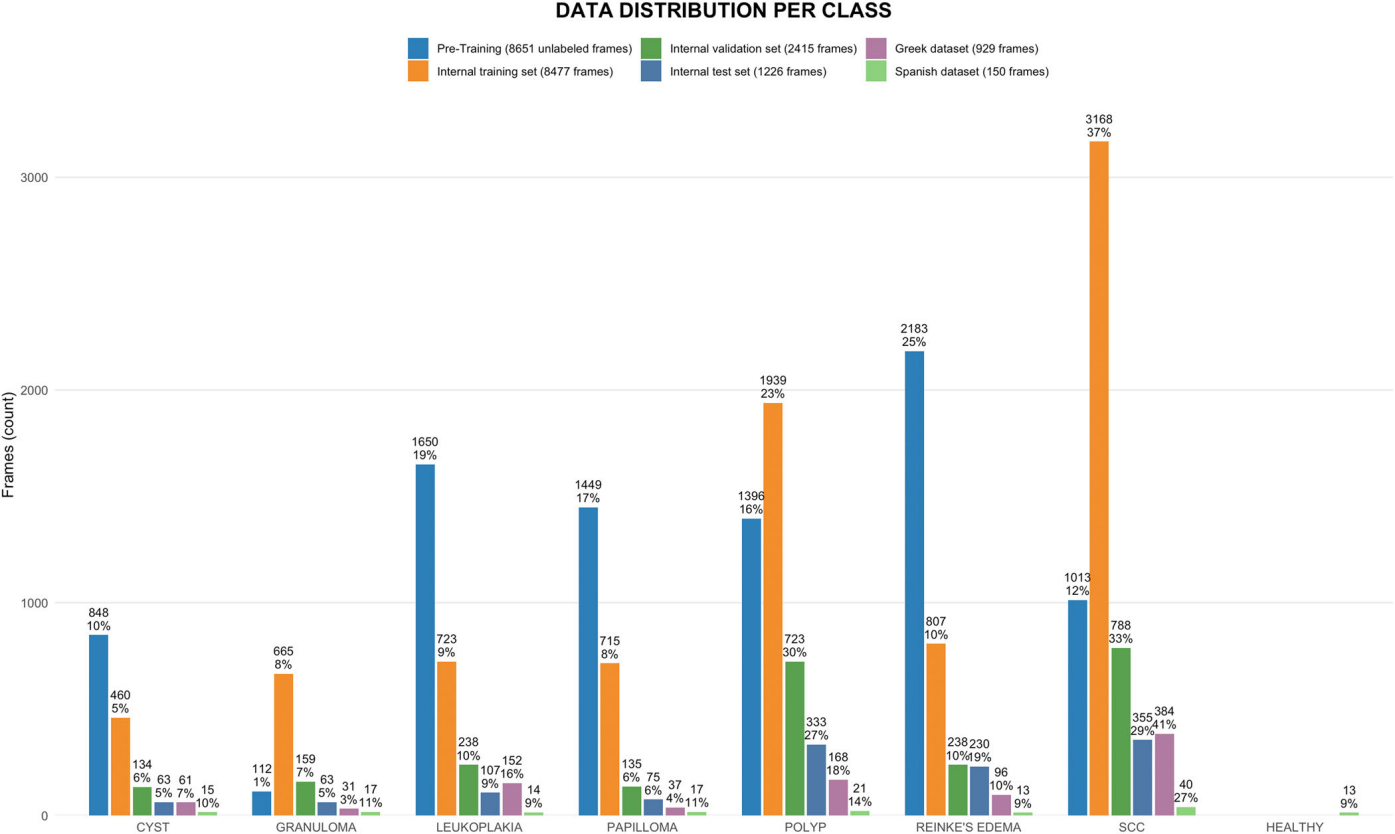
Overview of the datasets used in the study, divided according to the image categories. Percentages refer to each respective dataset.

A comprehensive breakdown of the number of frames allocated to each category within the *Internal* and *External* datasets is provided in Figure 3.

### CADx Model Performances on Internal and External Datasets

**Figure 4:**
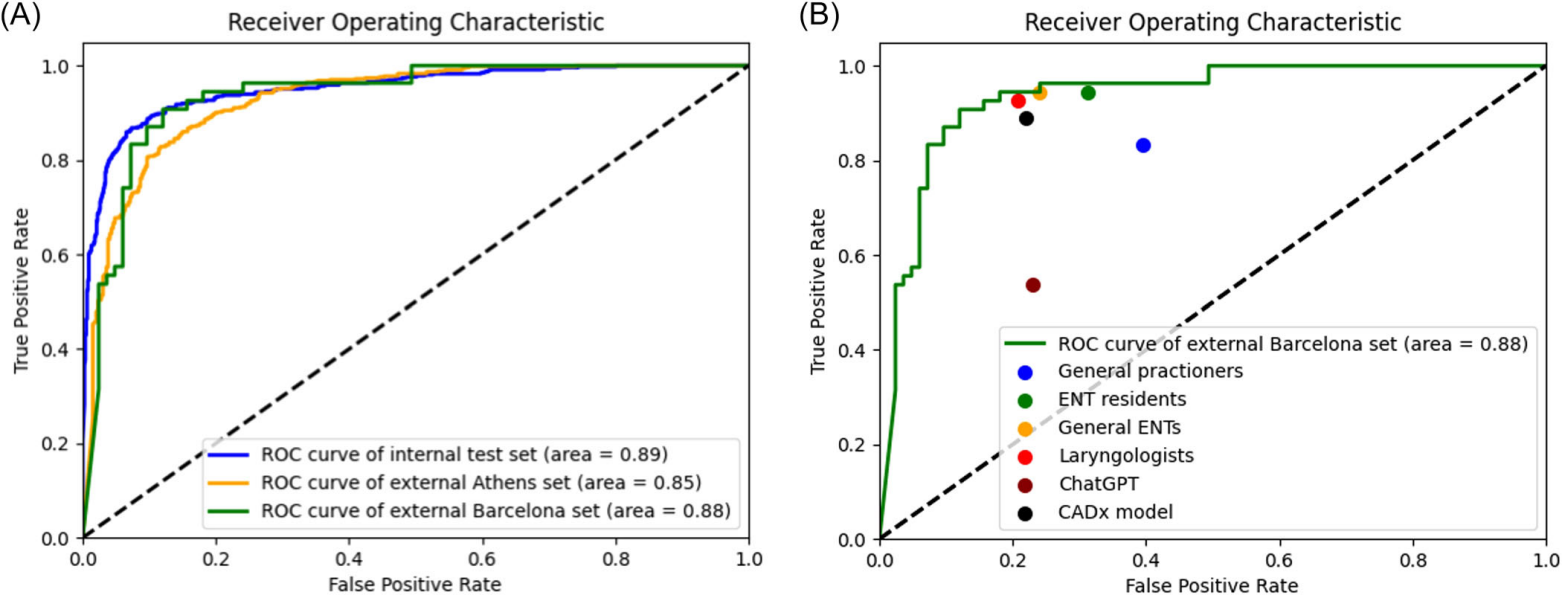
(A) Receiver operating characteristic (ROC) curves showing the CADx model performance on the 3 datasets. (B) Comparison between clinical raters, ChatGPT, and the CADx model.

On the *Internal* test set, the CADx model performed well, reaching an accuracy of 0.9 and an AUC of 0.89, respectively. On the *External* datasets, the model performed similarly with an accuracy of 0.85 and an AUC of 0.85 (*P* = .075) on the *Greek* dataset, and an accuracy of 0.88 and an AUC of 0.88 (*P* = .73) on the *Spanish* dataset Table 1 and Figure 4A).

**Table 1:** Performance Metrics Comparison Between the CADx Model Using the Internal Test Set and on the External Datasets (Greek Dataset and Spanish Dataset)

	Internal test set (n = 1226)			External *Greek* dataset (n = 929)			External *Spanish* dataset (n = 150)		
CADx model	Low‐risk	High‐risk	Weighted average	Low‐risk	High‐risk	Weighted average	Low‐risk	High‐risk	Weighted average
Precision	0.91 [0.87, 0.93]	0.9 [0.87, 0.92]	0.9 [0.88, 0.92]	0.79 [0.75, 0.83]	0.9 [0.87, 0.93]	0.86 [0.83, 0.88]	0.92 [0.86, 0.97]	0.83 [0.72, 0.93]	0.89 [0.83, 0.94]
Recall	0.94 [0.93, 0.96]	8.84 [0.80, 0.87]	0.9 [0.88, 0.92]	0.88 [0.84, 0.91]	0.83 [0.80, 0.86]	0.85 [0.80, 0.95]	0.88 [0.80, 0.95]	0.89 [0.81, 0.96]	0.88 [0.82, 0.93]
F1	0.92 [0.91, 0.94]	0.87 [0.84, 0.89]	0.9 [0.87, 0.92]	0.83 [0.80, 0.86]	0.87 [0.84, 0.89]	0.85 [0.83, 0.87]	0.9 [0.85, 0.95]	0.86 [0.78, 0.92]	0.88 [0.83, 0.93]
Accuracy	0.9 [0.87, 0.91]			0.85 [0.83, 0.87]			0.88 [0.83, 0.93]		
AUC	0.89 [0.87, 0.91]			0.85 [0.83, 0.88]			0.88 [0.83, 0.93]		

Abbreviation: AUC, area under the receiver operating characteristic curve. [95% confidence intervals].

### CADx Model Performance Comparisons With Clinical Raters and Large Language Models

**Figure 5:**
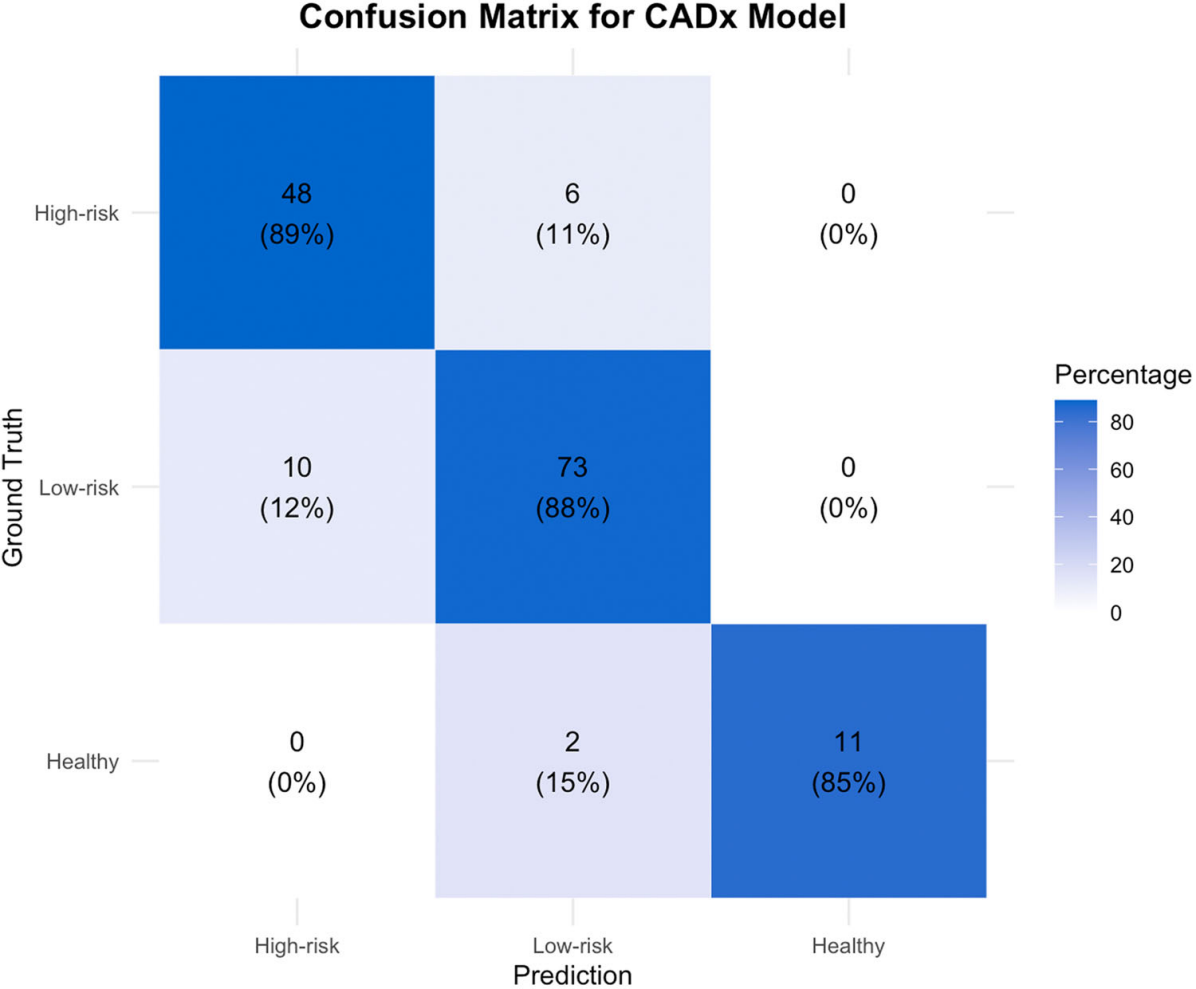
The CADx model confusion matrix. The true labels are on the vertical axis. The predicted labels are on the horizontal axis. The diagonal cells contain correctly classified instances, while off‐diagonal cells represent errors.

The comparison of the CADx model with the physicians and ChatGPT‐4o is summarized in Table 2. The model performed comparably to the otolaryngology residents (Accuracy 0.88. vs 0.87; *P* = .87), the general otolaryngologists (Accuracy 0.88. vs. 0.89; *P* = 1.00), and the expert laryngologists (Accuracy 0.88. vs 0.93; *P* = .28), while it performed significantly better in comparison to the general practitioners (Accuracy 0.88. vs 0.69; *P* < .001), and ChatGPT‐4o (Accuracy 0.88. vs 0.68; *P* = .001). Figure 4B depicts the ROC curve comparisons between the CADx model and the raters, while Figure 5 reports the confusion matrix of the CADx model. The confusion matrices of the clinical raters and ChatGPT‐4o are reported in Supplemental Figure S2, available online.

**Table 2:** Performance Metrics Comparison Between the CADx Model, the Physicians, and ChatGPT on the *Spanish* Dataset (n = 150)

		Precision			Recall			F1		
Rater	Accuracy	Healthy	High‐risk	Low‐risk	Healthy	High‐risk	Low‐risk	Healthy	High‐risk	Low‐risk
CADx model	0.88 [0.83, 0.93]	1.00 [1.00, 1.00]	0.83 [0.72, 0.92]	0.90 [0.83, 0.96]	0.85 [0.61, 1.00]	0.89 [0.79, 0.96]	0.88 [0.81, 0.95]	0.92 [0.76, 1.00]	0.86 [0.78, 0.92]	0.89 [0.84, 0.97]
General Practitioner	0.69 [0.63, 0.76] **(*P*** < **.0001)**	0.41 [0.21, 0.62]	0.64 [0.52, 0.76]	0.82 [0.73, 0.91]	0.69 [0.45, 0.93]	0.72 [0.60, 0.84]	0.66 [0.56, 0.76]	0.51 [0.29, 0.71]	0.68 [0.57, 0.77]	0.73 [0.65, 0.80]
Otolaryngology Resident	0.87 [0.81, 0.92] (*P* = .87)	0.93 [0.78, 1.00]	0.76 [0.65, 0.86]	0.96 [0.91, 1.00]	1.00 [1.00, 1.00]	0.94 [0.88, 1.00]	0.80 [0.70, 0.88]	0.96 [0.87, 1.00]	0.84 [0.77, 0.91]	0.87 [0.80, 0.92]
General Otolaryngologist	0.89 [0.84, 0.93] (*P* = 1.00)	0.79 [0.56, 1.00]	0.83 [0.74, 0.93]	0.95 [0.89, 0.99]	0.85 [0.62, 1.00]	0.93 [0.84, 0.98]	0.87 [0.79, 0.94]	0.81 [0.63, 0.95]	0.88 [0.81, 0.93]	0.91 [0.86, 0.95]
Laryngologist	0.93 [0.88, 0.97] (*P* = .28)	0.93 [0.78, 1.00]	0.89 [0.81, 0.96]	0.95 [0.90, 0.99]	1.00 [1.00, 1.00]	0.93 [0.85, 0.98]	0.92 [0.85, 0.97]	0.96 [0.87, 1.00]	0.91 [0.84, 0.96]	0.93 [0.89, 0.97]
ChatGPT	0.68 [0.61, 0.76] **(*P*** = **.0001)**	0.57 [0.30, 0.82]	0.68 [0.52, 0.82]	0.70 [0.61, 0.79]	0.62 [0.30, 0.86]	0.46 [0.33, 0.60]	0.83 [0.75, 0.91]	0.59 [0.31, 0.79]	0.55 [0.42, 0.67]	0.77 [0.68, 0.82]

The *P*‐values were calculated using McNemar's test comparing each rater's Accuracy (primary outcome) with the CADx model's value. *P*‐values in bold indicate statistical significance. [95% confidence intervals].

## Discussion

A recent review on AI in laryngology highlighted that current limitations in this field include, among others, a lack of large and diverse datasets, limited external validation, and generalizability issues.^18^ The authors suggested that collaborative, large‐scale studies with diverse datasets and robust validation methods are needed to overcome these barriers before AI can be fully integrated into laryngology practice. Indeed, many papers on CADx models in laryngology have recently been published.^14^, ^18^, ^19^ However, most of the studies relied on mono‐institution‐generated datasets, with only a few (24%) reporting some form of clinical comparison with humans, and with a lack of structured clinical validation.^18^ Moreover, almost none compared their results with external datasets. Our study is the first to incorporate both kinds of validations, which are necessary for a more comprehensive assessment of the clinical potential of AI models. Moreover, with more than 20,000 images, this study represents the largest multicenter trial focused on laryngeal disease classification in a non‐Chinese‐only cohort of patients.

Our network training method minimized the chances of overfitting, which is a main problem reported in previous studies,^20^ using a patient‐wise distribution of images between the training and test sets, ensuring that images of the same patients were not present simultaneously in both partitions. We also leveraged 2 datasets from 2 different centers for external validation. Moreover, our study is the first one to use multiple datasets from different regions of Europe and Asia, aiming to tackle possible ethnicity biases and strengthen the generalizability of the CADx model.

For the comparison task with human raters, it is important to specify that the few healthy cases (n = 13; 8%) available exclusively in the Barcelona dataset were considered outside the specific calculation of the AUC to allow unbiased comparisons with the other 2 test sets that did not contain healthy cases. Moreover, when one healthy case was flagged by a bounding box and submitted to the classification task, the error was attributable to the detection algorithm and not to the CADx model, which was trained only to distinguish between LR and HR. This explains why the only 2 healthy images that were flagged as having a lesion by the detection algorithm were classified as LR, because no high‐risk feature was present in the BB. As for the results on healthy images (confusion matrices in the supplemental information), it is interesting to note that both residents and laryngologists did not confuse any healthy image with other categories, while only one senior otolaryngologist misdiagnosed 2 healthy cases, similar to the CADx model. Finally, general practitioners performed the worst with 4 cases misdiagnosed.

Our results suggest that the CADx model's performance is not inferior to that of a general otolaryngologist. Only expert laryngologists outperformed the model, although not significantly. On the other hand, our CADx model outperformed general practitioners, otolaryngology residents, and the state‐of‐the‐art large language model ChatGPT, suggesting that rigorously training a specifically fine‐tuned deep learning computer vision model is currently the best strategy to pursue in this field.

These benchmark results are extremely important, as the implementation of automatic AI diagnosis solutions could be a game‐changer in global healthcare, especially for head and neck squamous cell carcinoma (HNSCC). In the near future, particularly in low‐income countries where HNSCC incidence is expected to rise, AI has the potential to transform cancer care by promoting more equitable access to early detection and treatment.^21^ AI‐assisted endoscopy could standardize the quality of screening examinations, reducing inter‐operator variability and enabling automated identification of high‐risk lesions through real‐time optical biopsy techniques. In limited‐resource settings, a CADx model such as ours could support timely referrals to specialized centers.^22^ Moreover, in settings where access to experienced specialists is scarce, AI‐powered diagnostic support tools can empower general practitioners and non‐specialist healthcare providers to perform effective screenings in underserved areas.

While AI presents immense promise, its successful integration into global HNSCC care will require robust validation through large‐scale, multicenter studies, as well as careful consideration of ethical issues such as algorithmic bias, and efforts to ensure accessibility to AI‐powered tools in low‐resource settings.^23^ Recent concerns have been raised about whether continuous exposure to AI might reduce the performance of standard non‐AI‐assisted endoscopy, suggesting a negative effect on endoscopist behaviour.^24^ However, we believe that well‐designed studies like ours can bridge this gap. The limitations of this study mainly consist of its retrospective proof‐of‐concept nature. Potential biases include the retrospective, multicenter design with convenience sampling of high‐quality frames from tertiary centers. Healthy images were excluded from primary metrics because their labeling depended on a preceding detection, which may result in partial verification and optimistic performance bias. Differing test setups (model on crops vs clinicians on full images/majority vote) also affect comparability. Moreover, this is a pilot study designed to assess whether the model can perform at a sufficient level before being implemented in real‐life clinical practice, and therefore was not fully conceived to evaluate the CADx model's clinical benefits. To assess the clinical benefits of this strategy, we are currently performing a prospective multicenter clinical trial to validate this AI‐assisted automatic diagnosis framework.

Our study represents a significant step toward the clinical translation of AI‐assisted lesion classification in laryngology. By incorporating both internal and external validation, leveraging a large multicenter dataset spanning diverse populations, and implementing robust strategies to mitigate overfitting, we provide the most comprehensive evaluation of a CADx model for endoscopic lesion classification to date. Our results demonstrate that the CADx model is robust and able to generalize well across different datasets, achieving accuracy comparable to general otolaryngologists.

Recognizing the need for future research to focus on real‐world implementation and the seamless integration of AI‐powered tools into clinical workflows for optimal patient care, this pilot study lays the groundwork for a multicenter prospective clinical trial evaluating the same model within real‐life clinical practice.

## Author Contributions

**Claudio Sampieri**, design the work, data acquisition, analysis, interpretation of data, drafting the work and reviewing it critically, and final approval; **Francesco Mora**, data acquisition, interpretation of data, drafting the work and reviewing it critically, and final approval; **Giorgio Peretti**, design the work, interpretation of data, drafting the work and reviewing it critically, and final approval; **Marc Larrosa Durá**, design the work, analysis, interpretation of data, drafting the work and reviewing it critically, and final approval; **Isabel Vilaseca**, design the work, interpretation of data, drafting the work and reviewing it critically, and final approval; **Francesc Xavier Avilés‐Jurado**, data acquisition, interpretation of data, drafting the work and reviewing it critically, and final approval; **Alessandro Ioppi**, design the work, interpretation of data, drafting the work and reviewing it critically, and final approval; **Elisa Bellini**, data acquisition, interpretation of data, drafting the work and reviewing it critically, and final approval; **Berta Alegre**, design the work, analysis, interpretation of data, drafting the work and reviewing it critically, and final approval; **Laura Ruiz‐Sevilla**, data acquisition, interpretation of data, drafting the work and reviewing it critically, and final approval; **Rakesh Srivastava**, data acquisition, interpretation of data, drafting the work and reviewing it critically, and final approval; **Athanasios C. Sakellaridis**, data acquisition, interpretation of data, drafting the work and reviewing it critically, and final approval; **Andriana Razou**, data acquisition, interpretation of data, drafting the work and reviewing it critically, and final approval; **Georgios P. Kotsis**, data acquisition, interpretation of data, drafting the work and reviewing it critically, and final approval; **Sara Moccia**, analysis, interpretation of data, drafting the work and reviewing it critically, and final approval; **Leonardo S. Mattos**, design the work, analysis, interpretation of data, drafting the work and reviewing it critically, and final approval; **Chiara Baldini**, design the work, analysis, interpretation of data, drafting the work and reviewing it critically, and final approval.

## Disclosures

### Competing interests

The views and opinions expressed herein are those of the authors alone and do not necessarily reflect those of the European Union or the European Commission.

### Funding source

C.S., F.M., G.P., I.V., S.M., L.D.M., and C.B. reported receiving grants from the European Union‐Next Generation EU‐ Horizon Program for the AIRCARE project.

## Supporting information

Supplemental Figure 1 TRIPOD + AI Checklist. This is a 27‐item checklist that aims to harmonize the landscape of prediction model studies and to provide transparent reporting of studies developing, validating, or extending (updating) a prediction model.
Supplemental Figure 2 The confusion matrices of the CADx model, clinical raters, and ChatGPT‐4o on the Barcelona external dataset. The actual labels are on the vertical axis, and the predicted labels are on the horizontal axis.

## Data Availability

The dataset and the related code are not publicly available because no ethical committee permissions were obtained for this purpose. No patients or members of the public were involved in the planning, design, conduct, reporting, or dissemination of the study and its findings.
