# Supplementary material for: Multicenter Clinical Validation of an Artificial Intelligence Diagnostic Classification Model for Laryngoscopy Images
Source: Otolaryngol Head Neck Surg. 2026 Feb 16;174(4):1049–58. doi: 10.1002/ohn.70153 (PMC13035010; doi:10.1002/ohn.70153)
Supplement: Supplementary file 1 — Supplemental Figure 1 TRIPOD + AI Checklist. This is a 27‐item checklist that aims to harmonize the landscape of prediction model studies and to provide transparent reporting of studies developing, validating, or extending (updating) a prediction model. Supplemental Figure 2 The confusion matrices of the CADx model, clinical raters, and ChatGPT‐4o on the Barcelona external dataset. The actual labels are on the vertical axis, and the predicted labels are on the horizontal axis. [file OHN-174-1049-s001.docx]

**Multicenter Clinical Validation of an Artificial Intelligence Diagnostic Classification Model for Laryngoscopy Images**

**Performance Metrics:**

-  Precision (also known as the positive predictive value): provides information about the model's ability to avoid false positives. It is unique to each category, meaning there is one independent precision value for each output class (HR, LR, Healthy).

$$Precision=\frac{\mathrm{TP}}{TP+FP}$$

-  Recall (also known as sensitivity): This metric calculates the percentage of objects correctly classified by the model compared to all the objects that should have been recognized. It is also unique to each class.

$$Recall=\frac{\mathrm{TP}}{TP+FN}$$

-  F1-score: this score represents the harmonic mean of precision and recall, which provides a comprehensive measure of the model’s diagnostic performance. Compared to Accuracy, it does not depend on the dataset distribution. Hence, it serves as a reliable diagnostic outcome, although it is also unique to each class

$$F1score=2\frac{\mathrm{Precision}\mathrm{Recall}}{Precision+Recal}=\frac{2TP}{2TP+FP+FN}$$

-  Accuracy. This metric evaluates the overall correctness of a model's predictions by measuring the percentage of correctly classified instances across the entire dataset. However, it can be significantly affected by class imbalance, which occurs when the different classes of the dataset are disproportionally distributed. Considering our test sets are evenly distributed, accuracy represents a robust measure and was therefore the primary outcome for assessing the overall diagnostic performance of the CADx model.

$$Accuracy=\frac{TP+TN}{TP+TN+FP+FN}$$

-Receiver Operating Characteristic (ROC) curve: This represents a probability curve that plots the TP rate (recall) against the FP rate. The closer the curve approaches the upper left corner, the higher the TP rate, the lower the FP rate, and the wider the Area Under the Curve (AUC). The AUC of a classifier provides an aggregate measure of performance.

**Supplemental Figure 1**


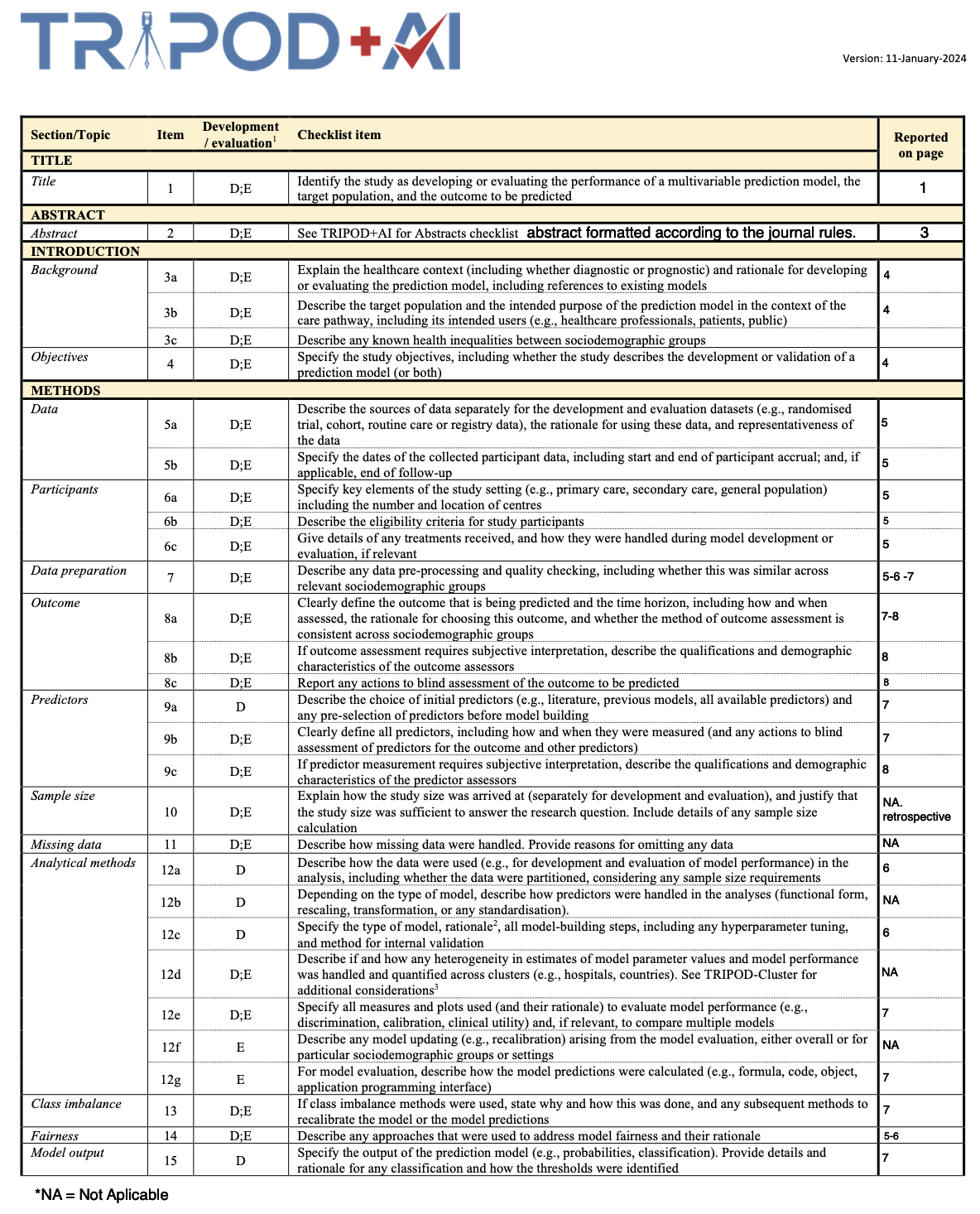


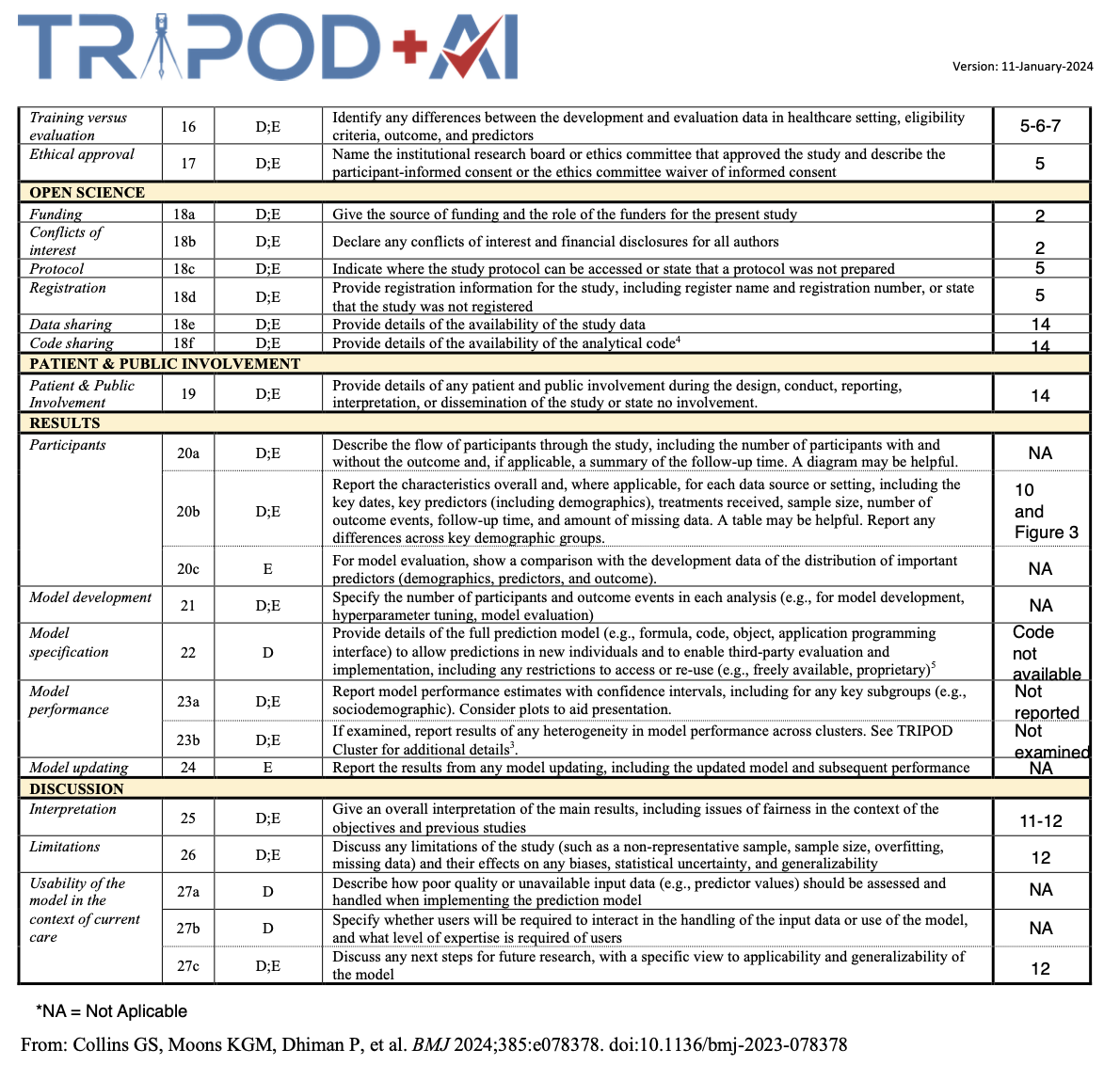


TRIPOD+AI Checklist

**Supplemental Figure 2**


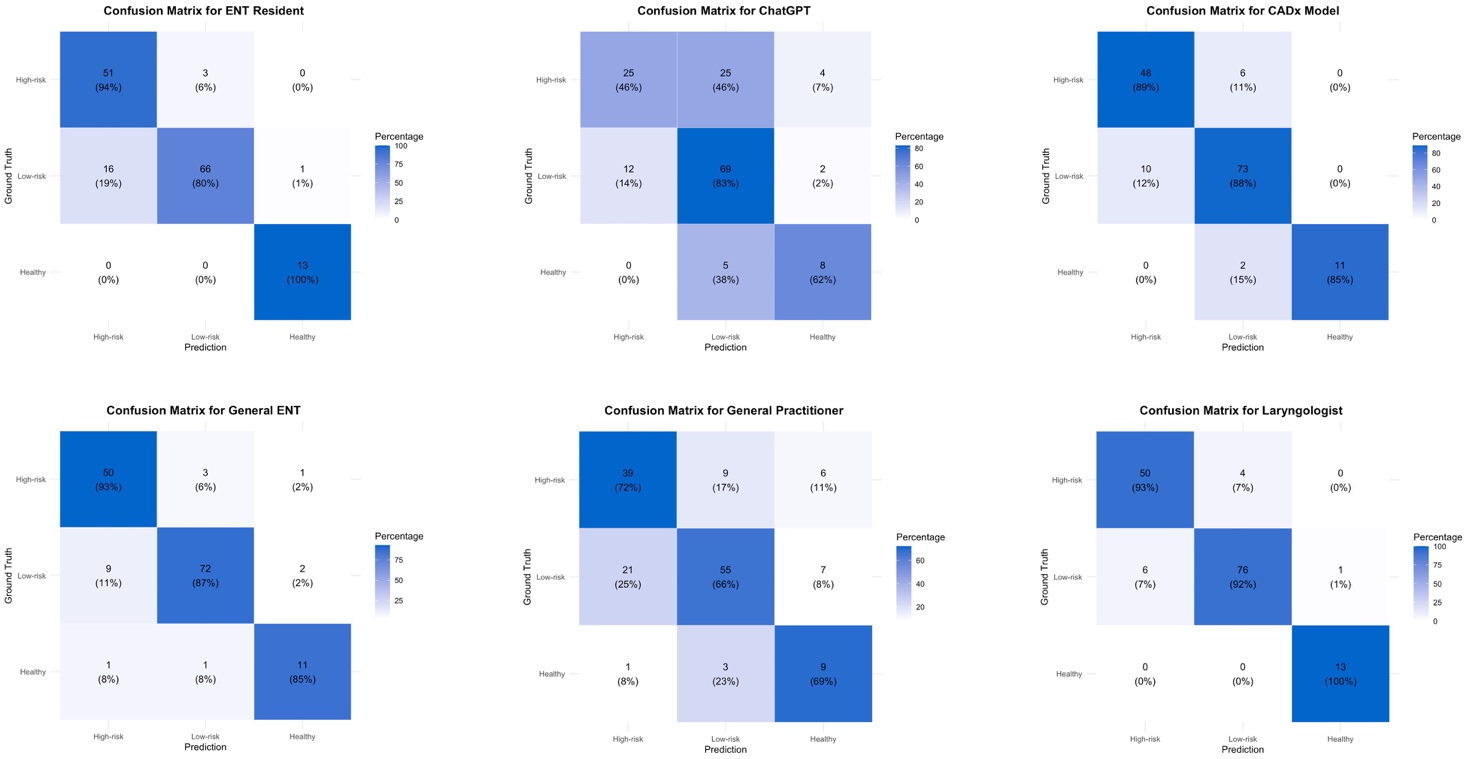


The confusion matrices of the CADx model, the 4 clinical raters, and ChatGPT-4o on the Barcelona external dataset. The actual labels are on the vertical axis, and the predicted labels are on the horizontal axis. The diagonal cells contain correctly classified instances, while the others contain errors.
